# Ptomaine Poisoning

**Published:** 1900-08-04

**Authors:** 


### "PTOMAINE POISONING."

While the official inquiry into the employment of the so-called "food preservatives" still drags its slow length along, the inquirers listening to the declarations of one expert that a grain of a particular preparation would be sufficient to produce acute dyspepsia, and to the declarations of another that a pound of it might be swallowed with absolute impunity, it is certain that medical practitioners have been called upon, especially since the commencement of the recent outburst of hot weather, in connection with which, in all probability, "preservatives" have been very freely used, to deal with quite a large number of scattered cases of the condition commonly described as "ptomaine poisoning." A person apparently in perfect health would be suddenly conscious of a general feeling of *malaise*, which would be followed, after the lapse of a few hours, by a definite and often somewhat sharp rigor, attended by the usual elevation of temperature, and terminated, in the most favourable examples, by abundant vomiting, and, if Dame Nature were left to her own devices, by a certain amount of diarrhœa. A restless and dream-troubled night would lead to a day of more or less disinclination for food and incapacity for business; and three or four of such days would probably elapse before recovery could be described as complete. In many instances, no cause for such an attack can be assigned, while in others it is attributed to some article of food which was regarded as suspicious when consumed, and which was consumed somewhere about twenty-four hours before the first appearance of the symptoms. These are clearly indicative of irritant poisoning; but the time required for their production seems to show them to have been due, not to the immediate and direct effect of anything which was swallowed, but to the subsequent multiplication of microbes which the anything in question must have contained. There can be no doubt about the poisoning, neither can there be any about the incubation period. When the persons who are attacked happen to have dined together, and especially when some of the attacks terminate fatally, there will be a public investigation as to the assumed causes of the disaster, and a considerable amount of public interest will be excited. It follows that what may be called the groups of cases usually become widely known and freely discussed, while those in which only single individuals are concerned, although of late they have been numerous, have attracted far less attention than they have deserved.

On a general review of these single cases, it seems by no means improbable that many food preservatives, although possibly innocuous in themselves, may be eminently injurious to the consumer by virtue of their effect in delaying the progress, or in masking the signs, of decomposition of a hurtful character. In an appeal heard recently at the Liverpool Quarter Sessions against the conviction of a grocer who had been fined £20, and costs, by the stipendiary magistrates, for selling margarine which contained 51 grains of borates to the pound, one analytical chemist deposed that a man would have to eat a ton of the mixture before he would suffer ill-effects, and another deposed that he "never knew a case where injury had been caused by the administration of boracic acid in any article of food." All this may be very true, but it does not touch the question. This is not whether boracic acid is injurious *per se*, but whether its presence may not conceal from the purchaser the commencement of decomposition of an injurious character. Assume a dealer to be in possession of a quantity of margarine, or of some other food, it matters not what, which betrays to his experience that it is on the verge of changes which will render it unsaleable. Assume him further to know that, although an ordinary dose of boracic acid would be ineffective, a full dose, say 51 grains to the pound, will have the effect of delaying further manifestations of change for a sufficient time to allow the whole of the suspected stuff to pass into the hands of the retail purchaser. Is it not practically certain in such a case that the ordinary dealer would apply the full dose of the "preservative," and would push the material into the ordinary channels of distribution with as little delay as possible? Is it not, to say the least, probable that the initial changes, so slight in their apparent character as only to display themselves to a trained observer, might nevertheless be due to the commencing activity of colonies of microbes which, if left to themselves, would speedily occasion manifest decomposition, and which, when merely checked in their growth and multiplication by boracic acid, would resume their mischievous activity as soon as they were placed under favourable conditions in the intestinal canal of a consumer? This seems to us to be the real danger in the supposed case, and also to be the probable explanation of the isolated examples of ptomaine poisoning, and it is an aspect of the question to which the attention of medical men and of the legislature may advantageously be directed.

